# The nervous system in the cyclostome bryozoan *Crisia eburnea* as revealed by transmission electron and confocal laser scanning microscopy

**DOI:** 10.1186/s12983-018-0295-4

**Published:** 2018-12-03

**Authors:** Elena N. Temereva, Igor A. Kosevich

**Affiliations:** Department of Invertebrate Zoology, Moscow State University, Biological Faculty, Leninskie Gory, 1-12, Moscow, 119991 Russia

**Keywords:** Cerebral ganglion, Tentacles, Lophophore, Evolution, Phylogeny

## Abstract

**Introduction:**

Among bryozoans, cyclostome anatomy is the least studied by modern methods. New data on the nervous system fill the gap in our knowledge and make morphological analysis much more fruitful to resolve some questions of bryozoan evolution and phylogeny.

**Results:**

The nervous system of cyclostome *Crisia eburnea* was studied by transmission electron microscopy and confocal laser scanning microscopy. The cerebral ganglion has an upper concavity and a small inner cavity filled with cilia and microvilli, thus exhibiting features of neuroepithelium. The cerebral ganglion is associated with the circumoral nerve ring, the circumpharyngeal nerve ring, and the outer nerve ring. Each tentacle has six longitudinal neurite bundles. The body wall is innervated by thick paired longitudinal nerves. Circular nerves are associated with atrial sphincter. A membranous sac, cardia, and caecum all have nervous plexus.

**Conclusion:**

The nervous system of the cyclostome *C. eburnea* combines phylactolaemate and gymnolaemate features. Innervation of tentacles by six neurite bundles is similar of that in Phylactolaemata. The presence of circumpharyngeal nerve ring and outer nerve ring is characteristic of both, Cyclostomata and Gymnolaemata. The structure of the cerebral ganglion may be regarded as a result of transformation of hypothetical ancestral neuroepithelium. Primitive cerebral ganglion and combination of nerve plexus and cords in the nervous system of *C. eburnea* allows to suggest that the nerve system topography of *C. eburnea* may represent an ancestral state of nervous system organization in Bryozoa. Several scenarios describing evolution of the cerebral ganglion in different bryozoan groups are proposed.

## Background

Bryozoans (= Ectoprocta) are marine colonial invertebrates comprising a phylum within Lophotrochozoa. The position of bryozoans on the phylogenetic tree of lophotrochozoans is still unclear. According to traditional view, Bryozoa is one of three groups (bryozoans, phoronids, and brachiopods) within the clade Lophophorata that includes animals with a lophophore – ciliated tentacle crown for filter-feeding [1–3]. Although the validity of the lophophorates has not been supported by most molecular studies [4–6], still there is some molecular [7, 8] and morphological [9, 11] evidence for the monophyly of the lophophorates. Else, according to other molecular data, Bryozoa constitutes the clade Polyzoa. This clade was defined in 2009 [5] and was recently supported by a molecular study [6].

The phylum Bryozoa includes more than 6000 species [12] that are divided onto three classes: Stenolaemata, Phylactolaemata, and Gymnolaemata (with the latter comprising the orders Ctenostomata and Cheilostomata). The relationships between the three main groups of bryozoans are generally well supported by both morphological and molecular studies [13]. Exclusively freshwater, non-calcified clade Phylactolaemata is the sister group to the clade uniting Stenolaemata and Gymnolaemata [14–17]. Within the latter, non-calcified Ctenostomata (comprising fresh- and brackish-water as well as marine forms) are traditionally considered to be ancestral to the remaining groups – Cheilostomata and Stenolaemata [18, 19], that are both exclusively marine and independently acquired calcified skeleton [18, 19]. Modern stenolaemates are represented by only one surviving clade, Cyclostomata.

Many aspects of the organization and development of Phylactolaemata and Gymnolaemata have been studied in detail [20]. In particular, the organization of the nervous system of adults and larvae has been investigated in a number species from both groups [21–34]. At the same time, data on the organization of cyclostome bryozoans are few. Their larvae and metamorphosis into ancestrula have been described just in several papers [25, 35–41]. Similarly, the structure of adult cyclostomes has been described in a few works only [42–48]. Information about the general anatomy of the nervous system and musculature is obtained by modern techniques for two different cyclostome species [49, 50].

In all bryozoans, the mouth and anus are located close to each other, and the digestive tract is U-shaped. In phylactolaemates and some ctenostomes, the cerebral ganglion is organized as neuroepithelium and contains internal cavity [26, 32, 33]. During development, the bryozoan ganglion is formed as a result of invagination of the epithelium of the forming gut [20]. Evolution of such a specific body plan and the cerebral ganglion in bryozoans remains unclear. Research of the nervous system in different cyclostome species may help to answer important questions about bryozoan evolution and bryozoan relationships with other lophotrochozoans.

The main goal of this paper is to describe the nervous system of the cyclostome *Crisia eburnea* and to discuss scenarios in the evolution of the nervous system in three main groups of Bryozoa.

## Results

### General morphology of *Crisia eburnea*

**Fig. 1:**
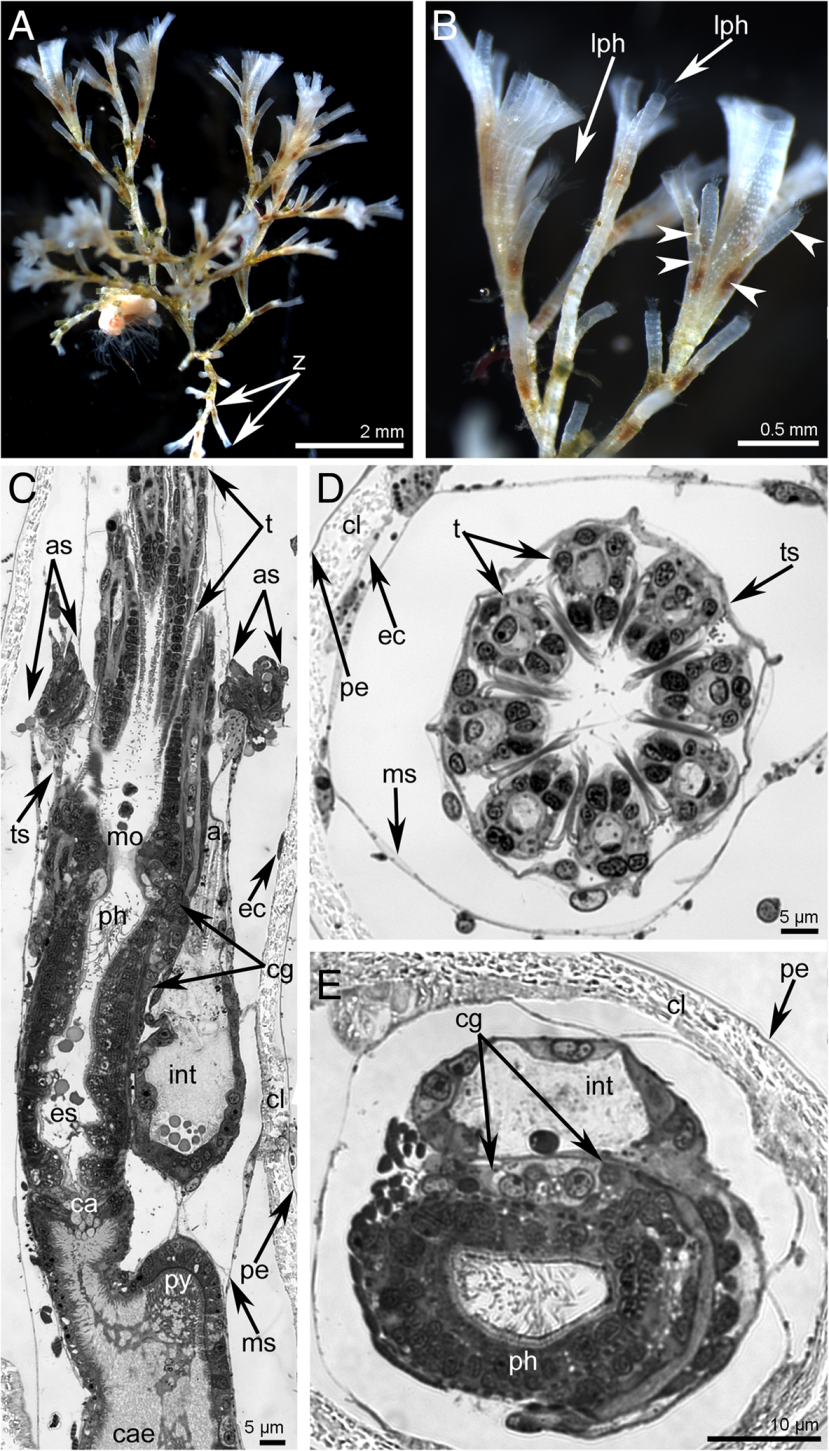
Organization of colony and zooids in *Crisia eburnea*. Photographs of live animals (**a**-**b**) and semithin sections (C-E). **a** A part of colony. **b** The same colony in higher magnification: tentacles and pores (arrowheads) in the wall of cystids are visible. **c** Sagittal longitudinal section of distal portion of zooid; tentacles semi-retracted. **d** Eight tentacles in the cross section. **e** Cross section of two branches of the digestive tract. Abbreviations: a – anus; as – atrial sphincter; ca – cardia; cae – caecum; cl – calcified layer; cg – cerebral ganglion; ec – epidermis; es – esophagus; int – intestine; lph – lophophore; mo – mouth; ms – membranous sac; pe – unmineralized cuticle; ph – pharynx; py - pylorus; t – tentacle; ts – tentacle sheath; z – zooid

Bush-like, branching colonies of *Crisia eburnea* consist of tubular zooids: anchoring rhizoids and feeding autozooids (Fig. 1a). Each autozooid comprises of a calcified body wall (cystid) and a soft polypide – a crown of eight tentacles (lophophore) associated with a gut. The lophophore is protruded via a terminal pore in the centre of the zooidal orifice covered by non-calcified terminal membrane (Fig. 1b). Pore leads to a small vestibulum, which walls continue to the walls of the tentacle sheath (or introvert). Chamber of the vestibulum continues to the atrium (cavity of the tentacle sheath) with a muscular atrial sphincter in the wall between these compartments. The cystid wall consists of an external unmineralized cuticle and calcified layer with epithelial monolayer underneath (Fig. 1c). The calcified layer bears numerous round gaps filled by cuticle plugs, i.e., pseudopores (Fig. 1b). Zooidal cavity is divided on the pseudocoel and coelom by the detached peritoneum – membranous sac. Its wall is very thin and consists of an outer basement membrane, annular muscles, and an inner peritoneal layer (Fig. 1c-e). When polypide is retracted, the tentacles are surrounded by the tentacle sheath (introvert). The wall of the tentacle sheath is composed of epithelial cell monolayer (which faces the tentacles), a basal membrane, longitudinal muscles, and an inner peritoneal lining (Fig. 1c). The U-shaped digestive tract consists of descending and ascending parts (Fig. 1e). The descending part begins by the central mouth surrounded by tentacles, following by the pharynx, esophagus, and stomach with cardia and caecum. The ascending part of the gut starts with a pylorus and followed by an intestine that opens by an anus (Fig. 1c).

### Cerebral ganglion

**Fig. 2:**
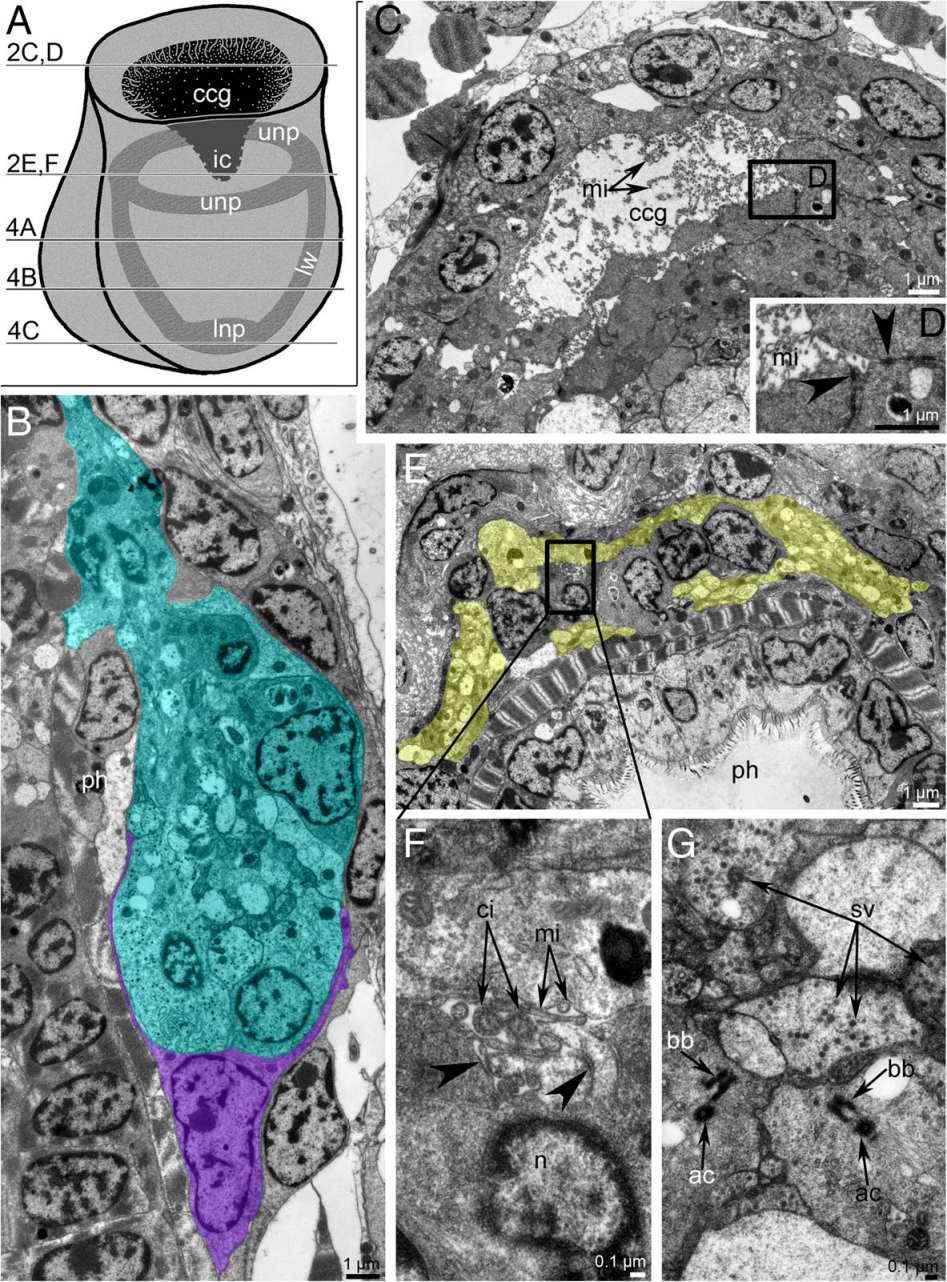
Organization of the cerebral ganglion of *Crisia eburnea*. **a** The scheme of the cerebral ganglion with upper concavity and neuropile inside. Different levels of the ultrathin sections are shown by number and letter on the left. **b** Longitudinal sagittal section of the cerebral ganglion (shown in cyan) and envelope cell (shown in magenta); TEM. **c** Cross section through the cavity in the upper part of the cerebral ganglion: the cavity is filled with thin microvilli. **d** A portion of the cavity: the microvilli and desmosomes (arrowheads) between cells are visible; TEM. **e** Cross section of the upper portion of neuropil (shown in yellow) surrounding several central perikarya; TEM. **f** Tiny cavity in the cerebral ganglion is filled with microvilli and cilia. Neurones are connected via desmosomes (arrowheads); TEM. **g** Cross section of the perikarya with basal body and accessory centriole; TEM. Abbreviations: ac – accessory centriole; bb – basal body; ccg – upper concavity of the cerebral ganglion; ci – cilia; ic – internal cavity; mi – microvilli; lnp – lower neuropil of the cerebral ganglion; lw – lateral weir of the cerebral ganglion; n – nucleus; ph – pharynx; sv – synaptic vesicle; unp – upper neuropil of the cerebral ganglion

**Fig. 3:**
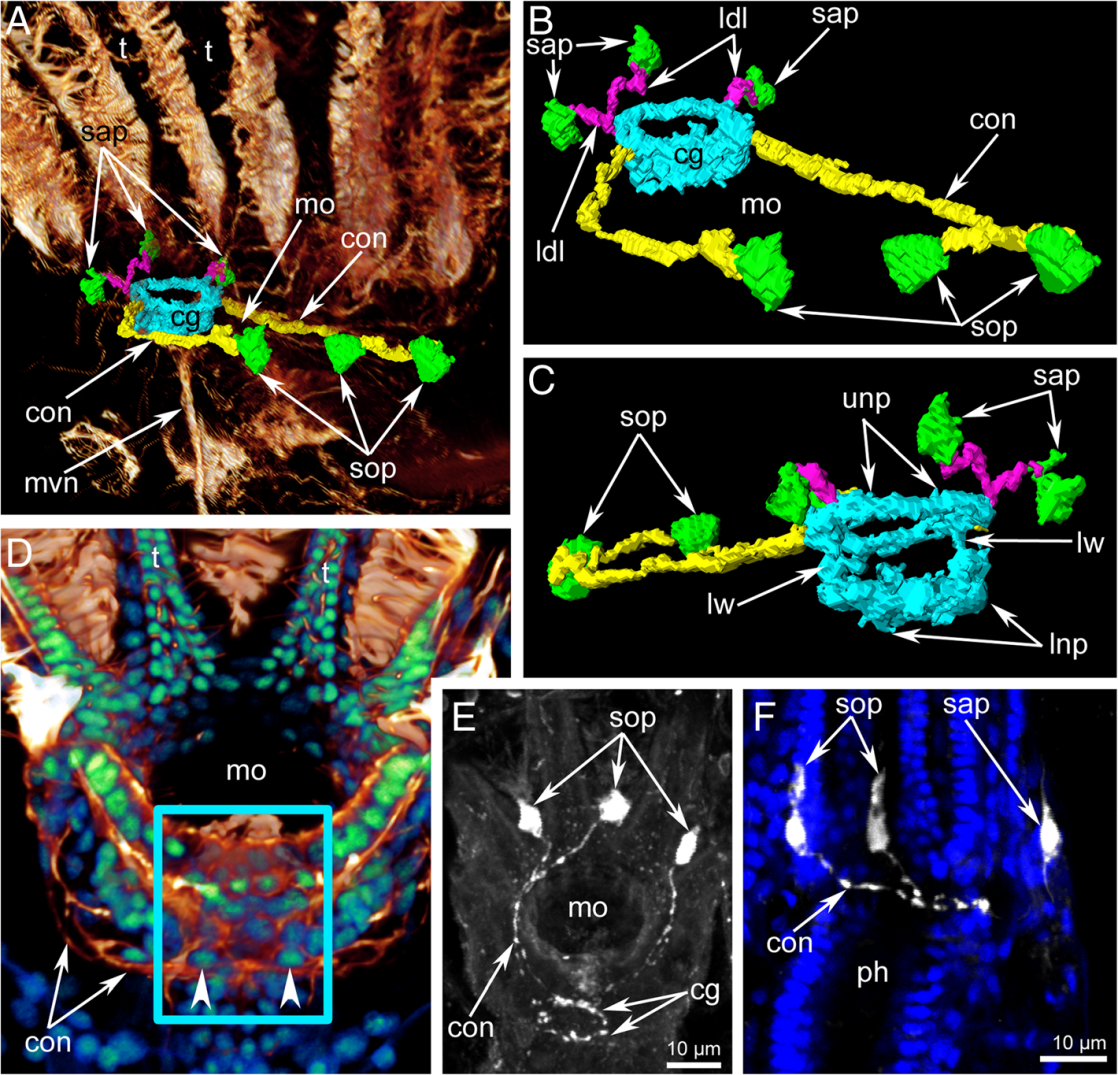
Serotonin-like immunoreactive nervous system of the lophophore in *Crisia eburnea* (CLSM)**.** 3D-reconstructions (**a-c**), volume rendering (**d**) (different parts of the serotonin-like nervous system shown by different colours), and Z-projections (**E-F**) of the lophophore after mono-, double, and triple staining for tyrosinated α-tubulin (glow/red), 5-HT (serotonin) (white), and DAPI (blue and green). **a** 3D-reconstruction combined with volume rendering. General view of serotonin-like immunoreactive nerve elements viewed from the right side. **b** Nerve elements viewed from the oral side. **c** Nerve elements viewed from the anal side. **d** Volume rendering of the lophophore viewed from the anal side. The area of the cerebral ganglion is framed by cyan rectangle. Two latero-distal perikarya are shown by arrowheads. **e** Serotonin-like perikarya and neurites of the lophophore viewed from the top. **f** Two oral and one anal large serotonin-like immunoreactive perikarya of the lophophore. Abbreviations: cg - cerebral ganglion; con – circumoral nerve ring; ldl – lophophoral anal nerves; lnp – lower neuropil of the cerebral ganglion; lw – lateral weir of the cerebral ganglion; mo – mouth; mvn – medial visceral nerve; ph – pharynx; sap – anal serotonin-like immunoreactive perikarya; sop – oral serotonin-like immunoreactive perikarya; t – tentacle; unp – upper neuropil of the cerebral ganglion

The cerebral ganglion is located on the anal side of the pharynx (Fig. 1e). The ganglion is bulb-shaped with a wide base and more narrow upper part (Fig. 2a, b). Immuno-staining against serotonin showed that the cerebral ganglion does not contain any serotonin-like immunoreactive perikarya, containing serotonin-like immunoreactive neurites that form a neuropil instead (Fig. 2a and Fig. 3a-c). The neuropil includes a circular upper portion and thickened basal portion; the upper and basal portions contact each other via two lateral weirs (Fig. 2a and Fig. 3c).

**Fig. 4:**
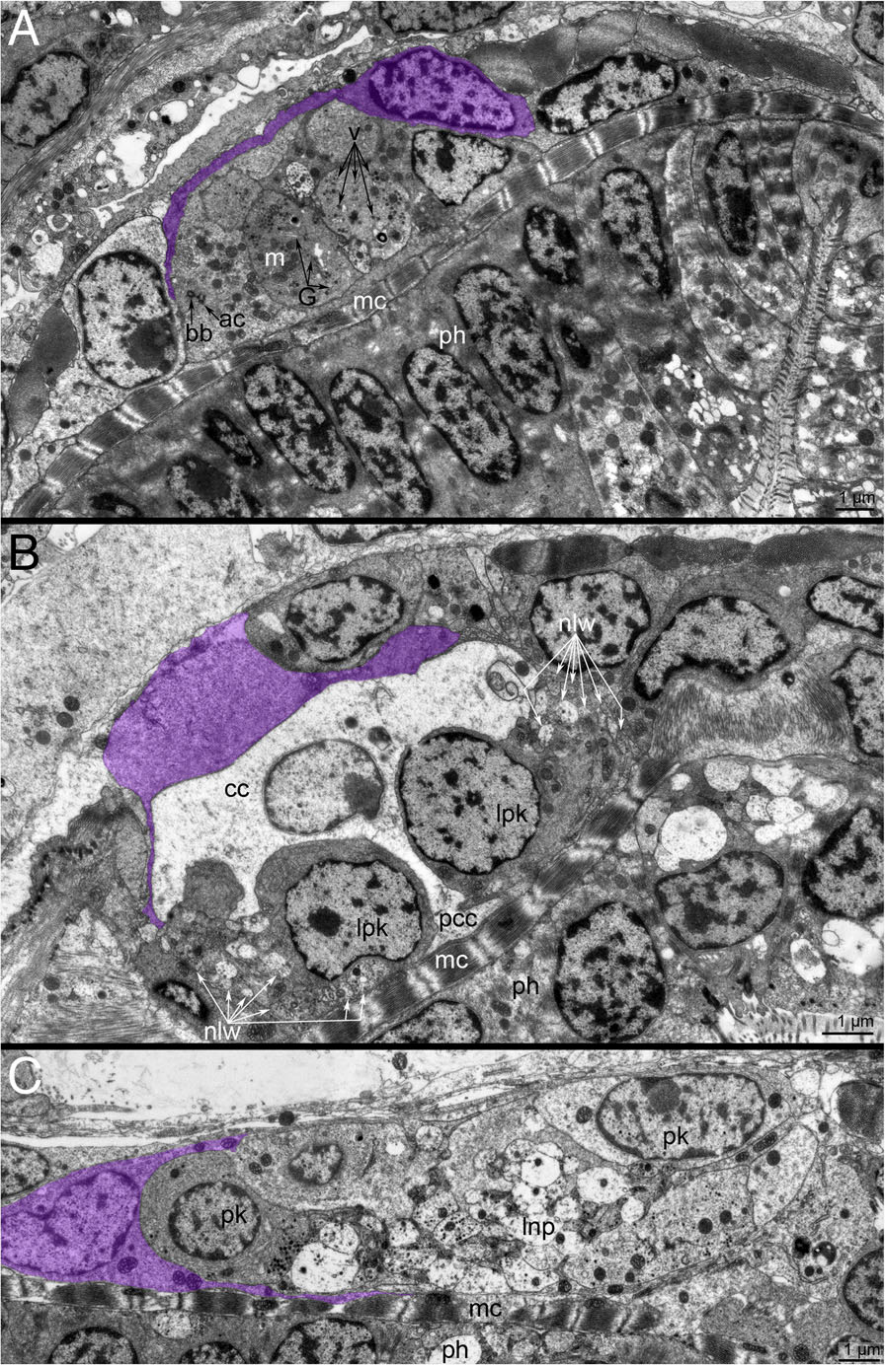
Organization the cerebral ganglion of *Crisia eburnea* at different levels (TEM). In all pictures, the envelope cells are marked by magenta. **a-c** Transverse thin sections. **a** The middle portion of the ganglion is formed by perikarya whose cytoplasm is filled with electron dense vesicles. **b** The lower portion contains two lateral perikarya that are associated with neurites of lateral weirs. **c** The lower portion of neuropil. Abbreviations: ac – accessory centriole; bb – basal body; G – Golgi apparatus; cc – central cell; lnp – lower neuropil; lpk – lateral perikaryon; m – mitochondria; mc – muscle cell of the pharynx; nlw – neurites of lateral weirs; pcc – projection of central cell; ph – cells of the pharynx; pk – perikaryon; v – vesicles with dense material

The ultrastructure of the cerebral ganglion differs at different levels (Fig. 2a). A wide concavity is located in the upper portion of the cerebral ganglion (Fig. 2a, c). This concavity is formed by cells that contact each other via desmosomes and whose apical surfaces form numerous thin branching microvilli, which fill the concavity (Fig. 2d). The upper part of the cerebral ganglion has the central inner area with perikarya that are surrounded by the neuropil (Fig. 2e). Else, the upper part of the ganglion contains a narrow cavity between neuropil and inner perikarya (Fig. 2f). The cavity is filled with microvilli and cilia that belong to perikarya, which surround the cavity (Fig. 2f). The cytoplasm of these perikarya contains a basal body and an accessory centriole (Fig. 2g). Further down the cerebral ganglion is formed by perikarya, which contain numerous mitochondria, Golgi apparatus, vesicles with electron-dense material, a basal body, and an accessory centriole (Fig. 4a). Below the latter cells, two lateral perikarya and one central cell are evident (Fig. 4b). The central cell is large, irregularly-shaped, and has electron-lucent cytoplasm. Each lateral perikaryon is associated with a group of longitudinal neurites, which probably form two lateral weirs between the upper and lower portions of the neuropil (Fig. 4b). These latero-distal perikarya can be also recognized within the cerebral ganglion by staining with DAPI and anti-tyrosinated α-tubulin (Fig. 3d). The lower part of the cerebral ganglion comprises numerous neurites of the lower portion of the neuropil, surrounded by a few perikarya (Fig. 4c). At different levels, the cerebral ganglion is surrounded by flattened envelope cells with thin, long projections that partly cover the perikarya and neurites (Fig. 2b and Fig. 4a-c).

### Nerve tracts projecting from the cerebral ganglion

**Fig. 5:**
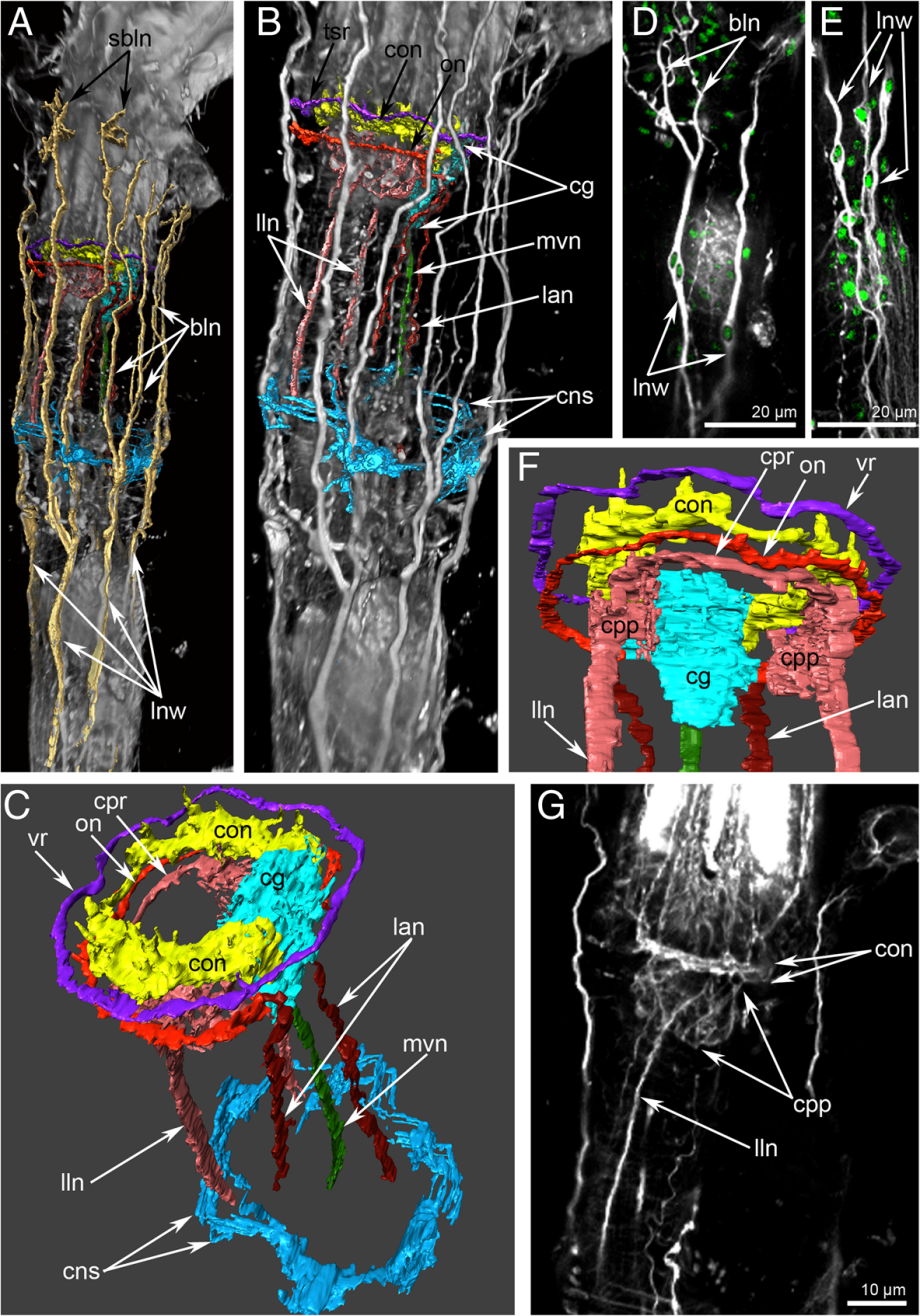
Main nerves of the zooid in *Crisia eburnea* (CLSM)**.** 3D-reconstructions (**a-c, f**), and Z-projections (**d-e, g**) of the polypide and cystid below a lophophore after mono- and double staining for tyrosinated α-tubulin (grey) and DAPI (green). Zooids with protruded (**a**-**f**) and retracted (**g**) lophophores are used. **a** 3D-reconstruction combined with volume rendering; view from the left side. The oral side is at the left. **b** The same reconstruction in higher magnification; the longitudinal nerves of the body wall are not indicated by abbreviations. **c** Main nerve elements of the lophophore base and cystid wall viewed from above. **d** Two branched longitudinal nerves of the body wall with nucleus in the centre of nerve fiber. (**E**) Longitudinal nerves of the body wall. **f** Main nerve elements of the lophophore base and cystid wall viewed from the oral side. **g** Left longitudinal lateral nerve of the digestive tract. Abbreviations: bln – branches of the longitudinal nerves of cystid; cg – cerebral ganglion; cns – circular nerves of atrial sphincter; con – circumoral nerve ring; cpp – circumpharyngeal nerve plexus; cpr – circumpharyngeal nerve ring; lan – latero-anal nerves; lln – longitudinal lateral nerve of the digestive tract; lnw – longitudinal nerves of the body wall; mvn – medial visceral nerve; on – outer nerve ring; sbln – small branches of the longitudinal nerves of cystid; vr – vestibular nerve ring

**Fig. 6:**
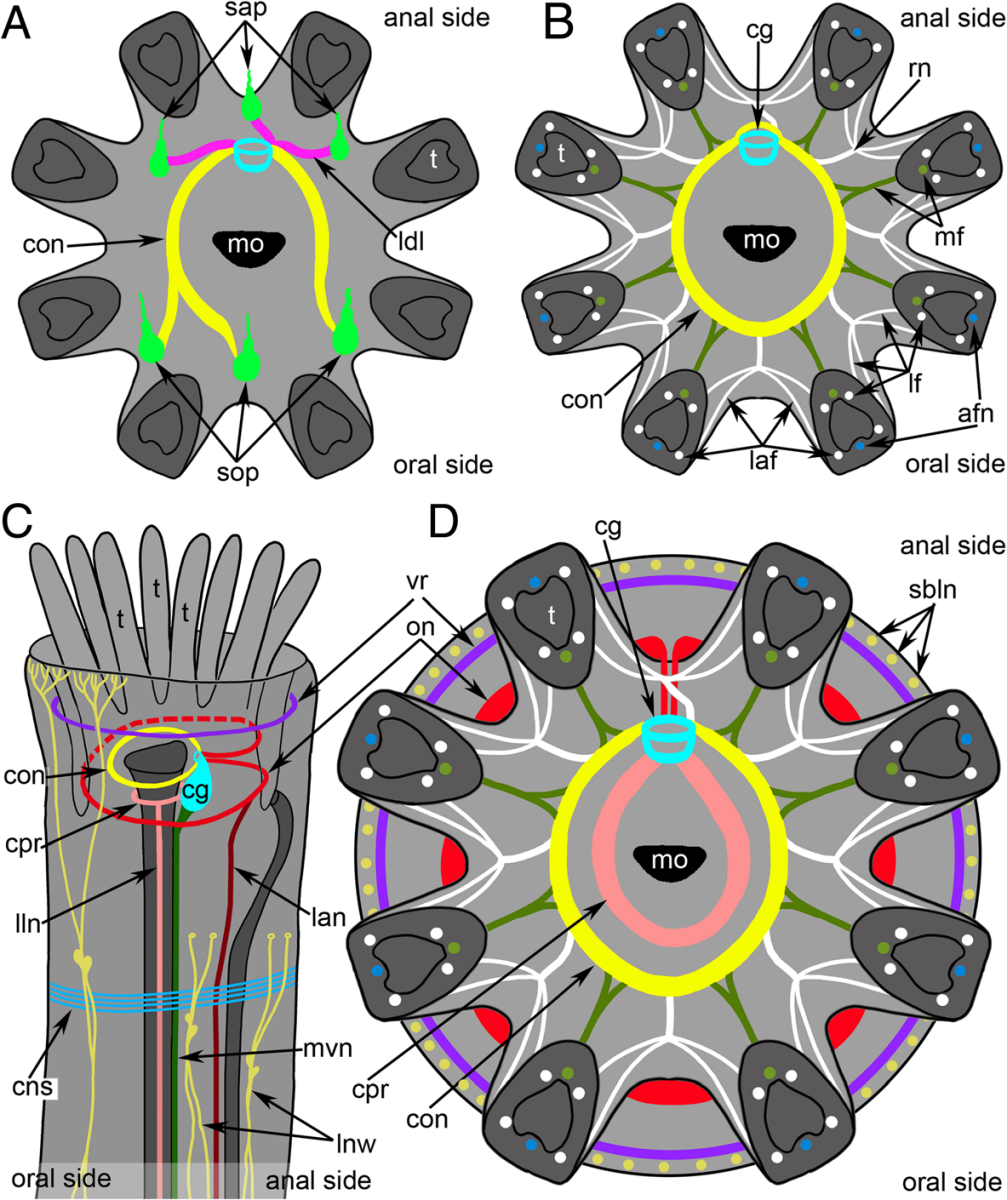
Schemes of the lophophore innervation in *Crisia eburnea*. **a** Organization of the serotonin-like immunoreactive nervous system of the lophophore, viewed from the top. **b** Innervation of tentacles. **c** The main nerve tracts of the lophophore and the body; lateral view. **d** Top view of the nerve elements after staining for tyrosinated α-tubulin. Abbreviations: afn – abfrontal tentacle nerve; cg – cerebral ganglion; cns – circular nerves of atrial sphincter; con – circumoral nerve ring; cpr – circumpharyngeal nerve ring; laf – latero-abfrontal tentacle nerve; lan – latero-anal nerves; ldl – lophophoral anal nerve; lf – laterofrontal tentacle nerve; lln – longitudinal lateral nerve of the digestive tract; lnw – longitudinal nerves of the body wall; mf – mediofrontal tentacle nerve; mo – mouth; mvn – medial visceral nerve; on – outer nerve ring; rn – radial (intertentacular) nerve; sap – anal serotonin-like immunoreactive perikarya; sbln – small branches of the longitudinal nerves of cystid; sop – oral serotonin-like immunoreactive perikarya; t – tentacle; vr – vestibular nerve ring

Immunostaining against serotonin and tyrosinated α-tubulin reveals several circular and longitudinal nerves, which extend from the cerebral ganglion (Figs. 3, 5a-c and Fig. 6a, c).

The circumoral nerve ring is the most prominent nerve tract originating from the circular upper neuropil of the cerebral ganglion and passing around the mouth (Fig. 3 and Fig. 5c). A few neurites of the circumoral nerve ring exhibit serotonin-like immunoreactivity (Fig. 3e, f). These neurites extend around the mouth but do not form a complete circle (Fig. 3a-c and Fig. 6a) and are associated with large serotonin-like immunoreactive perikarya, which are located at the base and between the oral tentacles (Fig. 3a-c, e, f and Fig. 6a). All studied specimens had two left and one right large, serotonin-like immunoreactive perikarya. They are flask-shaped with a thin apical part and a wide basal part, giving rise to the thin projection (Fig. 3f).

Three short nerves begin from the cerebral ganglion and extend to the anal side of the lophophore (Fig. 3a-c). There are two right anal nerves and one left nerve. Each anal nerve is associated with a large serotonin-like immunoreactive perikaron (Fig. 3a-c and Fig. 6a).

The circumpharyngeal nerve ring originates from the cerebral ganglion, extends around the pharynx, and looks like a thick nerve plexus with wide lateral portions and a narrow oral portion (Fig. 5f). The circumpharyngeal nerve ring gives rise to two lateral longitudinal nerves (Figs. 5b, c, f, g and Fig. 6c). Each lateral longitudinal nerve originates from the circumpharyngeal nerve ring as two branches, which then fuse and pass along the lateral side of the pharynx (Fig. 5g).

The medial visceral nerve extends from the basal portion of the cerebral ganglion and passes along the dorsal side of the descending portion of the digestive tract (Fig. 5c and Fig. 6c).

The outer nerve ring begins at the upper narrow part of the cerebral ganglion and going around the lophophore at the tentacle bases (Fig. 5c, f and Fig. 6c, d). On the anal side, the outer nerve ring gives rise to two latero-anal neurite bundles, which extend along the ascending portion of the digestive tract (Fig. 5c and Fig. 6c).

### Innervation of tentacles

**Fig. 7:**
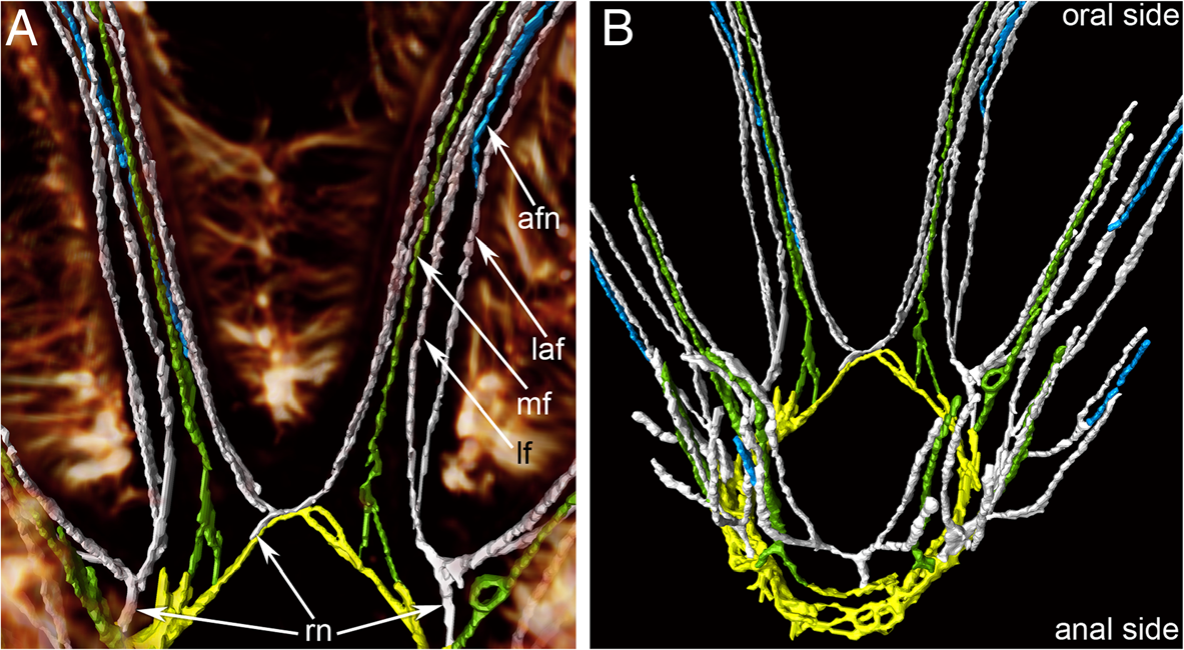
Innervation of tentacles in *Crisia eburnea* (CLSM)**.** 3D-reconstructions after staining for tyrosinated α-tubulin**. a** Nerves of two oral tentacles: 3D-reconstruction combined with volume rendering. The lateral cilia of tentacles are in glow/red. **b** Nerves of all tentacles; top view. Abbreviations: afn – abfrontal tentacle nerve; laf – latero-abfrontal tentacle nerve; lf – laterofrontal tentacle nerve; mf – mediofrontal tentacle nerve; rn – radial (intertentacular) nerve

**Fig. 8:**
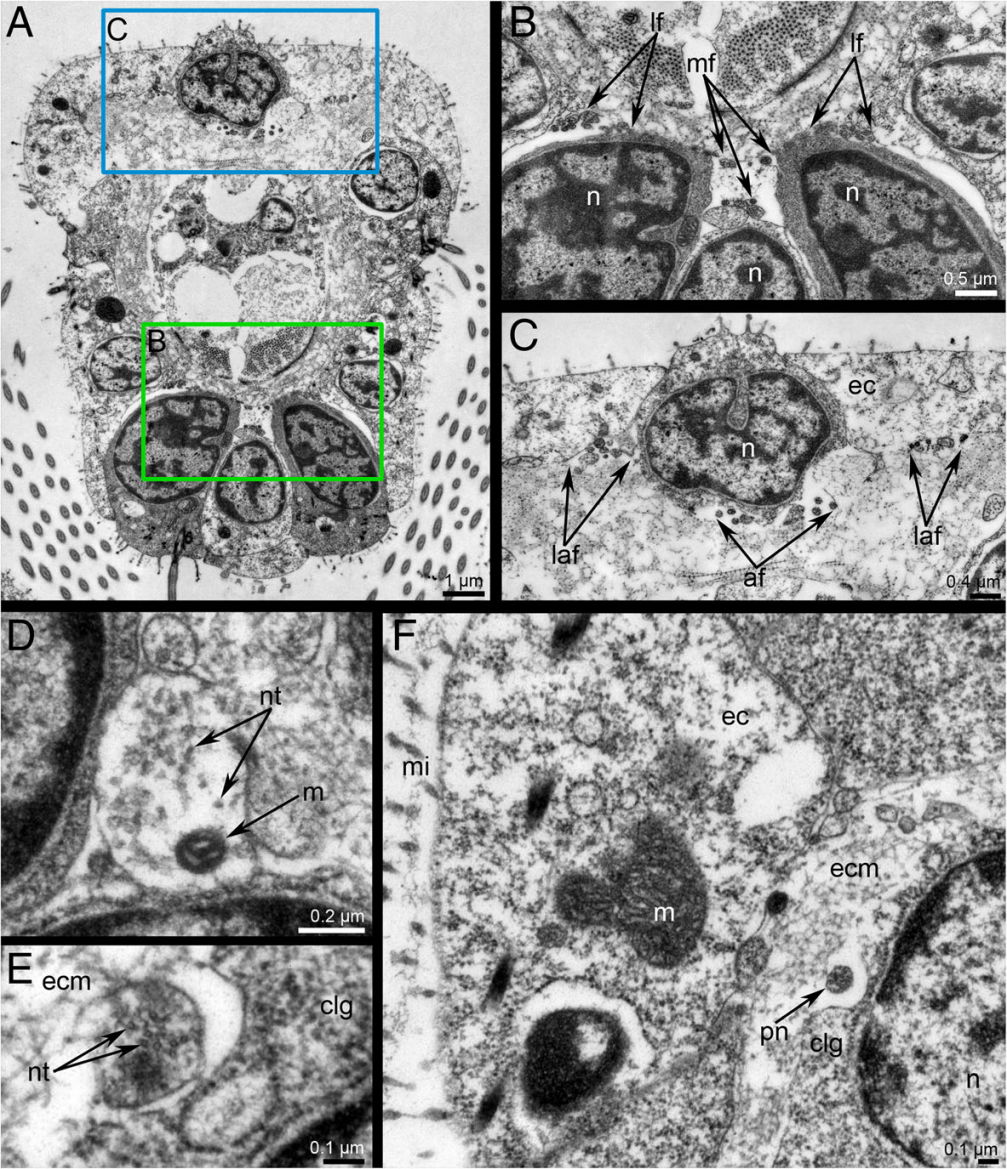
Innervation of tentacles in *Crisia eburnea* (TEM). Transversal ultrathin sections. **a** Whole tentacle; the abfrontal side is to the top. **b** Mediofrontal and laterofrontal neurite bundles. **c** Abfrontal and latero-anfrontal neurite bundles. **d** Neurite of large diameter of laterofrontal neurite bundle. **e** Peritoneal neurite. **f** Latero-abfrontal zone of tentacles with peritoneal neurite under the coelomic lining. Abbreviations: af – abfrontal tentacle nerve; clg – cell of coelomic lining; ec – epidermis; ecm – extracellular matrix; laf – latero-abfrontal tentacle nerve; lf – laterofrontal tentacle nerve; mf – mediofrontal tentacle nerve; m – mitochondrion; mi- microvilli; n – nucleus; nt – neurotubules; pn – peritoneal neurite

All tentacles are innervated from the circumoral nerve ring (Fig. 6b and Fig. 7a-b). Each tentacle contains six neurite bundles: one mediofrontal, two laterofrontal, one abfrontal, and two lateroabfrontal (Fig. 8a-c). The mediofrontal neurite bundles begin as two branches that fuse. The laterofrontal and lateroabfrontal neurite bundles extend from the intertentacular (radial) nerves. Each intertentacular nerve gives rise to two laterofrontal and two lateroabfrontal neurite bundles going into the adjacent tentacles (Figs. 6b and Fig. 7). Several microns from the tentacle base, the abfrontal neurite bundle arises from the lateroabfrontal neurite bundle (Fig. 7a, b and Fig. 8a, b). In some tentacles, abfrontal neurite bundle arises from the left lateroabfrontal neurite bundle, whereas in other tentacles, abfrontal neurite bundle arises from the right lateroabfrontal neurite bundle (Fig. 7a, b).

Tentacle nerves are basepithelial. According to TEM data, all tentacular neurite bundles are formed by 7–9 neurites whose diameter is about 100–120 nm (Fig. 8a-c). The neurites have electron-dense cytoplasm. The laterofrontal neurite bundles contain narrow neurites (about 100 mkm) with electron-dense cytoplasm and also thick neurites with large diameters (about 680–700 nm) and electron-light cytoplasm (Fig. 8d). Prominent longitudinal neurotubules are seen in the large neurites (Fig. 8d). Peritoneal neurites pass between coelothelial cells and the basal lamina (Fig. 8e-f). They are 150–300 nm in diameter, and their cytoplasm contains prominent longitudinal neurotubules (Fig. 8e).

### Nerves of the body, membranous sac, and digestive tract

**Fig. 9:**
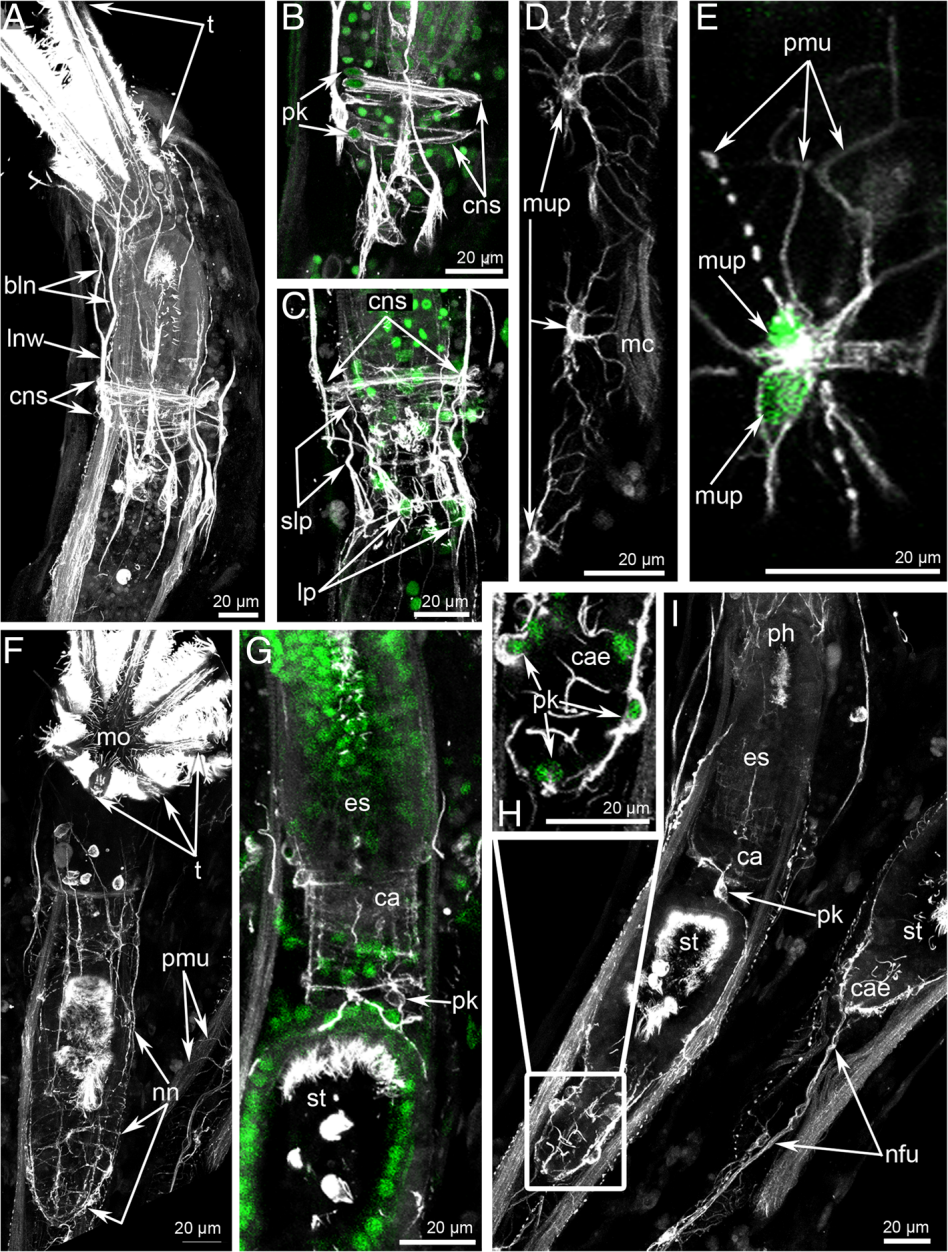
Innervation of the body wall, membranous sac, and digestive tract in *Crisia eburnea* (CLSM). Z-projections after mono- and double staining for tyrosinated α-tubulin (grey) and DAPI (green). Zooids with protruded (**a**-**f**) and retracted (**g**-**i**) lophophores are used. (**A**) The circular neurite bundle of atrial sphincter is located at level of cardia. Oral side is to the left. **b** Some nerve fibers with perikarya in the circular neurite bundle of atrial sphincter. Oral side is to the left; tentacles are to the top. **c** Large perikarya giving rise to the short longitudinal projections are associated with the circular neurite bundle. **d** Multipolar perikarya in the body wall. **e** Two closely contacted multipolar perikarya and their projections. **f** Nerve net of the membranous sac. **g** Perikarya and neurites of the cardia. **h** Perikarya around the caecum. **i** Some neurites and perikarya associated with cardia, caecum, and funiculus. Abbreviations: bln – branches of the longitudinal nerves of the body wall; ca – cardia; cae – caecum; cns – circular nerves of atrial sphincter; es – esophagus; lnw – longitudinal nerves of the body wall; lp – large perikarya; mc – muscle cells; mo – mouth; mup – multipolar perikarya; nfu – neurites of funiculus; nn – nerve net of the membranous sac; ph – pharynx; pk – perikarya; pmu – projections of multipolar perikarya; slp – short longitudinal projection; st – stomach; t - tentacles

Six thick longitudinal nerves extend along the body wall (Fig. 5a, b). In the upper-third of the zooid, each longitudinal nerve branches and gives rise to the two nerves that form numerous thin neurite bundles at the upper portion of the cystid wall (Fig. 5b). Each thick longitudinal nerve is formed by projections of two perikarya, whose nuclei are located in the center of the nerve (Fig. 5d, e). The upper-third of the zooid contains several (six-eight) circular nerves, which extend under the thick longitudinal nerves and form the nerve circle (Fig. 5b, c and Fig. 9a). Each circular nerve is formed by one neuron, whose perikaryon is visible on the oral side of the body (Fig. 9b). The circular nerves are associated with several large perikarya that form short longitudinal projections (Fig. 9c). Circular nerves and short longitudinal projections form a nerve grid in the upper-third of the zooid (Fig. 9c). The large multipolar perikarya are scattered along the wall of the cystid (Fig. 9d, e). Their processes contact each other forming a net (Fig. 9e). They also contact the longitudinal muscles of the cystid wall (Fig. 9d).

The membranous sac has a nerve net, which is formed by thin longitudinal and transversal neurite bundles (Fig. 9f).

The cardia is innervated by a few circular and longitudinal neurite bundles (Fig. 9g). Rare large perikarya are associated with the nerve net of the cardia (Fig. 9g, i). The caecum is mostly innervated by longitudinal neurite bundles (Fig. 9h, i).

## Discussion

### The organization of the cerebral ganglion

The anatomy of cerebral ganglion differs among the bryozoan species studied to date [45]. The most complex cerebral ganglion, consisting of a central and two lateral parts, is known in phylactolaemates [26]. In phylactolaemates, and gymnolaemates (cheilostomes and ctenostomes), the cerebral ganglion exhibits zonality [10, 42, 51]. This zonality is weak in phylactolaemates but prominent in ctenostomes and cheilostomes. In the cyclostome *Crisia eburnea*, the cerebral ganglion is characterized by presence of the upper and lower portions of the neuropil, variable cell composition and ultrastructure in its upper, middle, and lower parts. In addition, the cerebral ganglion contains two large lateral perikarya in this species, which could correspond to the large proximal perikarya in gymnolaemates [10, 42, 49]. In the cyclostome *Cinctipora elegans*, the cerebral ganglion is associated with two large groups of perikarya, which form the lateral ganglia [49]. Else, there is a large concavity in the upper portion of the cerebral ganglion. According to recent results [50], cerebral ganglion of *C. eburnea* consists of central part and two lobes and is associated with several subganglionic perikarya. Thus, in general, the cerebral ganglion of cyclostome bryozoans can be characterized as having concavity in the upper portion and additional perikarya in the lower portion.

The presence of the internal cavity is another peculiarity of the bryozoan cerebral ganglion. This cavity is prominent in phylactolaemates [26, 33], narrow in ctenostomes (when present) [10, 32], and is known in cheilostomes [51–53]. Since ganglion is formed as an invagination of the epidermal layer inbetween prospective mouth and anal areas during polypide development, its lumen is a former central part of this invagination [54, 55]. In *C. eburnea*, the large concavity is located in the upper portion of the cerebral ganglion. The small cavity, which is filled with microvilli and cilia, is presence in *C. eburnea*, but was not found in cyclostome *C. elegans* [49]. The detailed description of structure of the cerebral ganglion using transmission electron microscopy is required for those bryozoans that were studied by confocal laser scanning and light microscopy only.

**Fig. 10:**
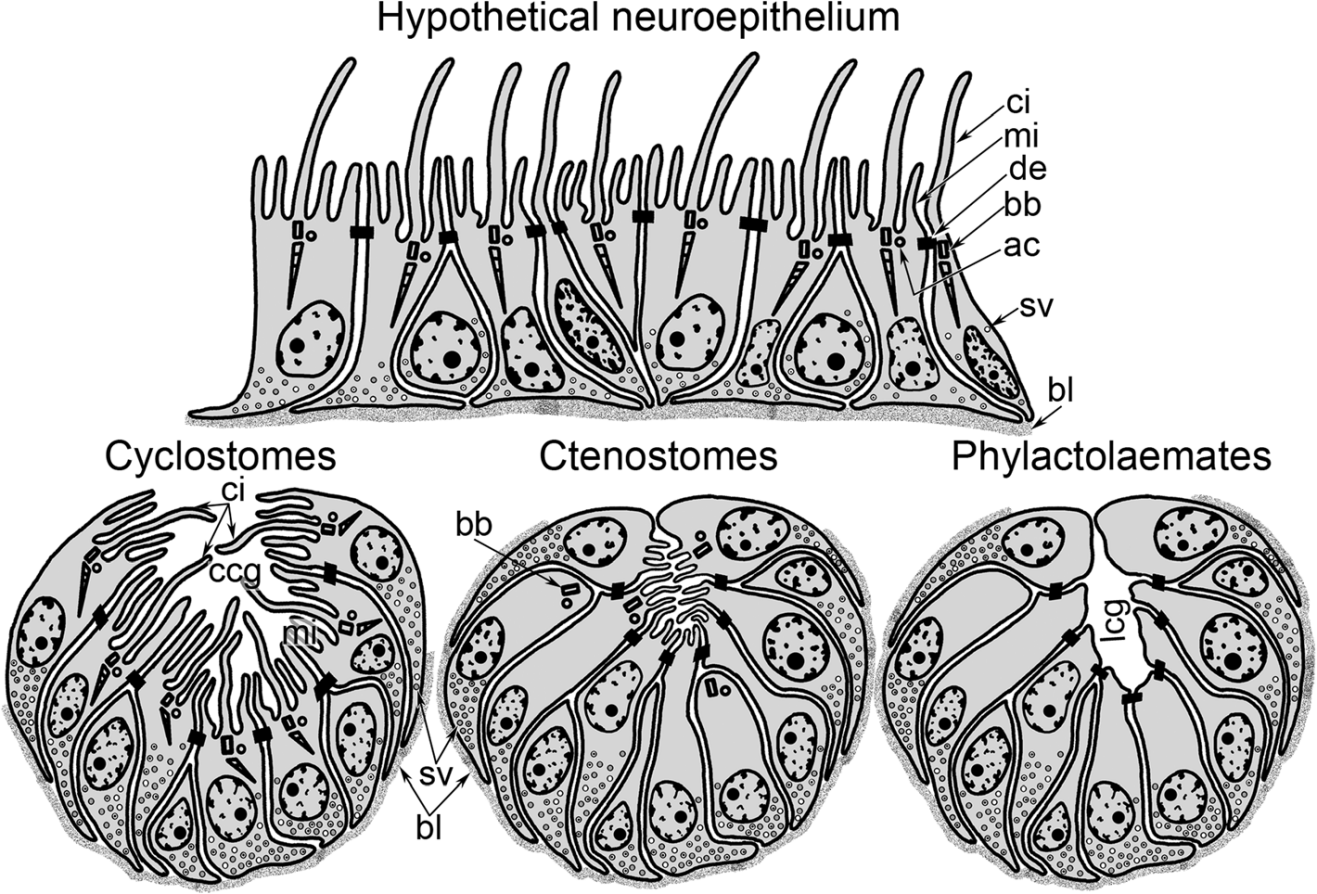
A possible scenario of the evolution of the cerebral ganglion. According to this scenario, a hypothetical neuroepithelium evolved into a concave ganglion in cyclostomates, and then into a ganglion with a small microvilli-filled lumen in ctenostomates, and finally into a ganglion with a prominent lumen in phylactolaemates. In cyclostomes, the ganglion retains cilia and microvilli; in ctenostomes, the ganglion lacks cilia but retains the basal bodies of the cilia and microvilli; and in phylactolaemates, the ganglion lacks both microvilli and cilia. In all cases, cells of the ganglion are connected by desmosomes. Abbreviations: ac – accessory centriole; bb – basal body; bl – basal lamina; ccg – concavity above the cerebral ganglion; ci – cilia; de – desmosome; lcg – lumen of the cerebral ganglion; mi – microvilli; sv – synaptic vesicles

The cerebral ganglion of *C. eburnea* exhibits features of neuroepithelium: there are desmosomes between the cells, and the cavity of the cerebral ganglion contains microvilli and cilia. Such an ultrastructure and the presence of a concavity in the upper portion of the ganglion suggests that the invagination of the cerebral ganglion shows evolutionary more primitive state in cyclostomes, whereas it is less primitive (while still possesssing microvilli in a lumen) in ctenostomes, and is advanced in phylactolaemates (Fig. 10).

According to our results, although some neurites of the cerebral ganglion of *C. eburnea* exhibit serotonin-like immunoreactivity, the perikarya in the cerebral ganglion lack such activity. Moreover, the cerebral ganglion of most bryozoans studied contains neither serotonin-like nor FMRFamid-like immunoreactive cells [10, 33]. FMRFamide- and serotonin-like immunoreactive perikarya have been found in the cerebral ganglion or in nerves near the ganglion only in *Cristatella mucedo* [33] and *Amathia gracilis* [10]. Recent result revealed several serotonin-like immunoreactive central perikarya in the cerebral ganglion of *C.eburnea* [50]. Further extensive research is required to show the pattern of distribution of these neurotransmitters in Bryozoa.

### Nerves projecting from the cerebral ganglion

In all bryozoans, the most prominent nerve extending from the cerebral ganglion is the circumoral nerve ring [10, 31, 32, 49, 50]. The connection of the circumoral nerves and the large serotonin-like immunoreactive perikarya situated near the tentacle bases has been described for all bryozoans studied to date. According to most authors, the number of large serotonin-like immunoreactive perikarya is equal to the number of tentacles minus 2, and this is also applied to *C. eburnea*. The specific function of these perikaya is unclear: in most bryozoans, they are not connected to the tentacular nerves and do not apparently contribute to the tentacle innervation.

Among bryozoans, cheilostomes have exactly three oral perikarya, whereas phylactolaemates and some ctenostomes have more than three oral perikarya. Based on this characteristic, the nervous system of cyclostomes *C. eburnea* ^[herein]^, [50] and *C. elegans* [49] looks similar to that of cheilostomes. An unusual feature of *C. eburnea* is the asymmetry in the position of the left and right perikarya in the oral and anal sides.

In some papers, the circumoral nerve ring is named as circumpharyngeal nerve ring [32], but the most interesting thing is the presence of two (circumoral and circum-pharingeal) or one (circumoral) nerves around the upper portion of the oral part of digestive tract. In all ctenostome bryozoans studied to date, the circum-pharyngeal nerve ring is represented by a thick and voluminous nerve plexus [10, 32, 49]. Circumpharyngeal nerve ring has not been described in both cheilostomes and phylactolaemates [30, 33, 34]. Although some phylactolaemates have a pharyngeal plexus that includes a pair of prominent longitudinal pharyngeal neurite bundles, the structure does not form a ring [33]. In the cyclostome *C. eburnea*, the anatomy of the circumpharyngeal nerve ring is similar to that of ctenostome bryozoans. According to recent results, lower pharynx perikarya are associated with the pharynx of *C. eburnea* [50]. Although the circumpharyngeal nerve ring is not described in *C. elegans*, the strong anti-tubulin-like immunoreactivity is evident around the foregut of this species [49].

In ctenostomes and cheilostomes, the circumpharyngeal nerve ring usually connects to three longitudinal nerves of the gut: two laterovisceral nerves that pass along the dorsolateral sides of the pharynx and one mediovisceral nerve that extends along the dorsal side of the pharynx [10, 32, 49, 51]. In *C. eburnea*, the circumpharyngeal nerve ring connects to the two lateral longitudinal nerves but does not connect to the mediovisceral nerve, which extends fron the cerebral ganglion directly. The same is described in *C. elegans* [49].

The presence of the outer nerve ring is described in two cyclostome species studied to date: *C. eburnea* and *C. elegans* [49]. Among bryozoans, the outer nerve ring was first found in the ctenostome *Amathia gracilis* [10], and is apparently homologous with the tentacle nerve ring of phoronids and with the lower brachial nerve of brachiopods [55]. The presence of an outer nerve ring in both ctenostomes and cyclostomes again raises the questions of the homology of the lophophore and of the monophyly of the lophophorates [9, 10, 56].

### Innervation of tentacles

Innervation of tentacles in *C. eburnea* has more in common with phylactolaemates since they both have six longitudinal tentacular nerves. Moreover, in both *C. eburnea* and phylactolaemates, tentacular nerves have the similar origination: the laterofrontal and lateroabfrontal tentacular nerves extend from the intertentacular (=radial) nerves [33, 34, 50]. In contrast, gymnolaemates have four longitudinal nerves in each tentacle: one frontal, two laterofrontal, and one abfrontal [32, 54]. Intertentacular nerves have also been described in gymnolaemates, but they give rise to the laterofrontal and abfrontal tentacular nerves [31, 49].

The presence of six neurite bundles in each tentacle of *C. eburnea* contradicts the early information about the presence of only four tentacular neurite bundles in the same species [42] and is also inconsistent with data on innervation of tentacles in cyclostome *C. elegans* [44]. In order to resolve these contradictions, the innervation of tentacles should be studied by TEM in other species of cyclostome bryozoans.

The presence of peritoneal neurites is characteristic of the tentacular nerve system of all lophophorates: these neurites have been described in phoronids [56–58], brachiopods [9, 55, 59], and bryozoans [10, 32, 49, 60]. Peritoneal neurites have not been described in the tentacles of other coelomic bilaterians, and their presence in all lophophorates makes innervation of the tentacles a consistent characteristic of this group.

### Innervation of the body, membranous sac, and digestive tract

In most bryozoans, the nerve elements of the body wall are represented by thin neurites and scattered perikarya, which form the nerve plexus [20, 22, 32, 33]. An unusual feature of the nervous system of *C. eburnea* is the presence of thick longitudinal nerves that pass along the body. Such nerves have not been described in other bryozoans. The presence of these thick longitudinal nerves probably correlates with the presence of longitudinal ectodermal muscles, which extend along the cystid wall and which were detected at the young ancestrula stage [40] and in adults of *C. eburnea* [50]. This accompaniment of thick muscles (retractors) with neurite bundles was previously described in the phylactolaemate *Hyalinella punctata* [33]. The distal ends of the longitudinal nerves dichotomously are supposedly branch in the wall of introvert above the atrial sphincter with circular nerves. Large multipolar perikarya, which innervate the cystid, are described in *C. elegans* and *C. eburnea*. These perikarya form a nerve net, which probably innervates the musculature of the cystid. Further TEM-study is needed to make these details more clear.

Accordingly to new data on myoanatomy of *C. eburnea* [50], circular nerves, which were described here for the first time, innervate musculature of the atrial sphincter. In prtotruded zooids, the atrial sphincter is located at level of cardia, where the circular nerves were detected.

The innervation of the bryozoan digestive tract has been studied in several researches [21, 33, 34, 50]. A detailed description has been published only for the phylactolaemate *H. punctata* [34]. Innervation of pharyngeal ridges was described in *C. eburnea* [50]. In this bryozoan, the digestive tract is innervated by several longitudinal nerves and numerous circular nerves, which together form a nerve plexus. Some circular nerves connect with sensory cells, which are embedded in the epithelium of the digestive tract [33, 50]. In gymnolaemates, the digestive tract is innervated by several longitudinal nerves that extend along the anal side [10, 31]. In *C. eburnea*, a thick nerve plexus, which includes neurites and perikarya, develops around the pharynx, cardia, and caecum. The presence of a visceral nerve plexus in *C. eburnea* is consistent with the inference that a diffuse neural plexus is part of the ground pattern of the Bryozoa [34].

## Main results and conclusions

The presence of the narrow internal cavity with cilia in the cerebral ganglion of *C. eburnea* allows to suggest that the anatomy of the cerebral ganglion in this species is the most primitive among all bryozoans.

Nervous system in the cyclostome *C. eburnea* exhibits both phylactolaemate and gymnolaemate features. First, the innervation of *C. eburnea* tentacles has more in common with the innervation of phylactolaemate tentacles, because there are six tentacular nerves in species from both groups. On the other hand, the frontal neurite bundles originate from the circumoral nerve ring in *C. eburnea* and in gymnolaemates but originate from intertentacular (radial) nerves in phylactolaemates. Second, the innervation of the intestine is plexus-like in *C. eburnea* and in phylactolaenates. On the other hand, *C. eburnea* has a nerve ring around the pharynx, which is typical for gymlolaemates, but not for phylactolaemates. Third, the membranous sac in *C. eburnea* is innervated by a diffuse plexus, which is typical for phylactolaemates, but the body wall is innervated by several main longitudinal nerves.

The presence of primitive-like cerebral ganglion and the combination of plexus-like and cord-like patterns of the nervous system in the membranous sac and body wall of *C. eburnea* allows to suggest that it represents rather primitive state of the nervous system organization in the Bryozoa. We suggest that cyclostomes could inherit a primitive plexus in their membranous sac from the ancestor with nerve plexus like in Phylactolaemata. Subsequently, the longitudinal lateral nerves of the body wall are the secondary modification. In turn, this plexus disappeared in modern gymnolaemates that acquired longitudinal nerve elements in their wall independent of cyclostomes.

## Materials and methods

### Sampling of animals and light microscopy

Material was collected in the vicinity of N.A. Pertsov White Sea Biological Station of Lomonosov Moscow State University (Kandalaksha Bay) (66°34′ N, 33°08′ E). Specimens of *Crisia eburnea* (Linnaeus, 1758) growing on red algae were collected in July 2017 by SCUBA diving at 7–15 m depth. Live animals were photographed using a Leica DFC420 (5.0MP) digital camera mounted on a stereo light microscope Leica M165C. Prior to fixation, bryozoans were anesthetized overnight in a solution of 5% MgCl2*6H_2_O and filtered sea water (1:1) at 8 °C. Zooids with expanded lophophores and retracted polypides were studied.

### Immunocytochemistry

Animals were fixed with 4% paraformaldehyde (PFA; Fluka, Germany) in phosphate-buffered saline (PBS; Fluka, Germany) at 4 °C for 24 h. Afterwards the fixed material was washed three times for 1 h in PBS and three times for 1 h in permeabilisation solution containing 0.1% Triton-X100 (Ferak Berlin, Germany), 0.05% Tween 20 and 0.1% NaN3 (Sigma) in PBS (PBT). Then the specimens were decalcified for 12 h in 5% EDTA in PBS at 8 °C. After decalcification material was washed for 5 h in PBT. Zooids with expanded lophophores were cut off the colony and processed further.

For immunocytochemical staining specimens were treated with blocking solution (1% BSA, 0.1% cold fish skin gelatin (Sigma), 0.5% Triton X-100, 0.05% Tween 20, 0.05% sodium azide in PBS)(BS) three times for 6 h following 85 h incubation at 4 °C with desired primary antibodies diluted in BS. Primary antibodies used were anti-serotonin (rabbit polyclonal, 1:1000; Chemicon, Temecula, CA, USA), and anti-tyrosinated α-tubulin (mouse monoclonal, 1:1600; Sigma, USA). After washing four times for 12 h in BS, the bryozoans were incubated 85 h at 4 °C with 1:500 dilution of Donkey Anti-Rabbit IgG Antibodies labeled with Alexa Fluor 647 (Molecular Probes, #A10040) and Donkey Anti-Mouse IgG Antibodies labeled with Alexa FluorR 546 (Molecular Probes, #A21202).

After washing three times for 4 h in PBS the material was blocked with 3% BSA in PBS for 10 h. Further on the specimens were incubated for 18 h in DAPI nuclei stain (100 ng/ml; Sigma) and BODIPY FL Phallacidin (488) (1:100, # B607, Molecular probes). Subsequent to washing for 5 h in PBS specimens were mounted on a cover glass covered with poly-L-lysine (Sigma-Aldrich, St. Louis, MO, USA) and embedded in Murray Clear.

Negative controls included specimens processed without incubation in primary antibodies. Autofluorescence control was prepared without addition of fluorochrome (secondary antibodies).

Specimens were investigated with Nikon A1 confocal microscope (Tokyo, Japan) (White Sea Biological Station, Russia). Optical longitudinal sections were obtained with a 0.5–1 μm step size.

Z-projections were generated using the program ImageJ version 1.43. 3D reconstructions were done in Amira version 5.2.2 software (Thermo Fisher Scientific, MA, USA). TEM micrographs and Z-projections were processed in Adobe Photoshop CS3 (Adobe World Headquarters, San Jose, California, USA).

### Tramsmission electron microscopy

For electron microscopy the animals were fixed overnight at 4 °C in 2.5% glutaraldehyde in phosphate buffer saline with addition of NaCl (pH 7.4, Osmolarity 830 milliosmols) [48]. Afterwards the fixed material was washed three times for 1 h in the same buffer saline and decalcified for 12 h in 5% EDTA in PBS at 8 °C. Decalcified material was washed three times for 1 h in phosphate buffer saline. Zooids with expanded lophophores were cut off the colony and postfixed for 2 h in 1% osmium tetroxide in the same buffer saline. After washing with the phosphate buffer saline the specimens were transferred through ethanol series and stored in 70% ethanol at 4 °C. Further preparation included dehydration in ethanol series and acetone, and embedding in Epon-Araldite resin (Electron Microscopy Sciences, Fort Washington, PA, USA). Semithin and thin sections with thickness of 0.5 μm were cut with Leica EM UC6 ultratome (Leica, Germany). Semithin sections were stained with methylene blue, observed with Zeiss Axioplan2 microscope and photographed with an AxioCam HRm camera. Ultrathin sections were stained in uranyl acetate followed by lead nitrate and examined with JEM-1011 JEOL and JEM-100 B-1 JEOL transmission electron microscopes (JEOL, Akishima, Japan). For TEM, four zooids from different branches of two different colonies were serially cut. Ultrathin sections were done every 2 μm. Ten ultrathin sections from different levels were studied for each specimen.
